# Localization-based full-field microscopy: how to attain super-resolved images

**DOI:** 10.1038/srep12365

**Published:** 2015-07-23

**Authors:** Taehwang Son, Wonju Lee, Donghyun Kim

**Affiliations:** 1 School of Electrical and Electronic Engineering Yonsei University, Seoul, 120-749 Korea

## Abstract

In this study, we have investigated localization-based microscopy to achieve full-field super-resolution. For localized sampling, we have considered combs consisting of unit pulses and near-fields localized by surface nanoapertures. Achievable images after reconstruction were assessed in terms of peak signal-to-noise ratio (PSNR). It was found that spatial switching of individual pulses may be needed to break the diffraction limit. Among the parameters, the resolution was largely determined by sampling period while the effect of width of a sampling pulse on PSNR was relatively limited. For the range of sampling parameters that we considered, the highest resolution achievable is estimated to be 70 nm, which can further be enhanced by optimizing the localization parameters.

Many super-resolution microscopy techniques have been developed to exploring molecular interactions in diverse environments by reducing optical point-spread function (PSF) or based on stochastic emission of fluorescent molecules^1^,^2^,^3^,^4^. A significant part of the super-resolution microscopy techniques take advantage of localization of light fields to achieve image resolution below diffraction limit. The localization based microscopy attempts to extract image information by sampling target fluorescence with super-localized light fields that are much smaller than the diffraction limit and reconstructing the sampled data with localization-specific deconvolution algorithms. The possibility of subdiffraction limited resolution was shown using metamaterials^5^, through statistical anaylsis^6^, with an sCMOS camera^7^, and for particle localization in 3D^8^,^9^,^10^. Various superlenses have been proposed in the related context and developed for various applications^11^: for example, by way of the coupling between evanescent diffracted wave and propagating Bloch wave of photonics crystals^12^ and by super-oscillation of a band-limited wave induced by diffraction grating^13^. Also, random nanoislands were used to observe internalization of virus and transmembrane protein perfusion^14^,^15^. Plasmonic localization of light wave based on simple nanoapertures was applied to fluorescence sampling for super-resolved molecular information^16^,^17^,^18^. Arrays of nanopillars were utilized to excite small focal volumes for subdiffraction-limit fluorescence imaging^19^. On the other hand, axial distribution of fluorescently tagged molecules was sampled by the localized field based on extraordinary optical transmission (EOT)^20^.

An ultimate goal of these approaches is to provide super-resolved full-field images. Oftentimes, however, requirements on the way that the fields are localized to attain the goal have not been clearly understood. Also, the implication of field localization on the acquired image has not been thoroughly studied. In this study, we intend to address these issues to find the conditions on localized fields for super-resolved image reconstruction and to investigate possible distortion in the reconstructed images in case that the conditions are not satisfied. The approach described here is applicable to localization microscopy that relies on super-localized sampling grids working in the near-field, regardless of specific techniques that may be used for localization of near-fields, and can be extended into other imaging modalities, for example, based on surface-enhanced Raman spectroscopy.

For this purpose, we first make a quick review the Nyquist-Shannon sampling theorem based on a delta comb. Although sampling itself is a topic that has been developed for many decades, it has been little explored in the context of localization-based super-resolution microscopy^21^. In this sense, effects of a sampling comb are examined when an equally ideal, yet more practical, unit pulse is employed to excite fluorescence. The results will then be extended to sampling based on surface-enhanced near-fields that are localized by nanoaperture arrays. For simplicity, following assumptions have been made in the analysis:

(AS1) The imaging system is linear space-invariant.

(AS2) Near-field distribution is known to an arbitrary spatial precision in 3D.

(AS3) Fluorophores are labeled in saturation with molecular density exceeding the Nyquist criterion and size effect of fluorophores is ignored.

(AS4) Photons that are fluorescently excited from localized fields are incoherent with each other.

(AS1) simply states that the linear imaging theory holds^22^ Therefore, intensity distribution in the image plane can be described by the optical transfer function (OTF) of an imaging system and spatial translation in the object plane does not affect the point-spread function. (AS2) is critical for microscopy that depends on near-field distribution. However, from an experimental perspective, near-field distribution is often measured with an insufficient precision especially when extremely high detection sensitivity is simultaneously required^23^, and this can set the limit on the achievable resolution. (AS3) assumes that fluorescent particles are much smaller than localized near-fields, which is satisfied in most practical circumstances. Although the fluorescent intensity distribution may be affected by several factors including molecule orientations respect to the local field components^24^, the distribution can thus be assumed to follow that of near-fields in ensemble-averaged fluorescence excitation^25^. (AS3) and (AS4) state that the fluorescent emission distribution in the near-field, which eventually gives rise to an image, is spatially correlated to the evanescent near-field that excites fluorescence. If, for example, fluorescent particles are so large that the size effect may not be ignored and depending on the fluorescent emitter states, the emission distribution takes a complex point spread and cannot be identical to the near-field distribution^26^. Effects of probe size and labeling density on the imaging characteristics such as spatial resolution are described elsewhere^27^.

## Theoretical Backgrounds

### Nyquist-Shannon sampling based on delta comb

The Nyquist-Shannon sampling theorem (see Appendix A for details) states that exact reconstruction of an object is possible if the object is band-limited such that its Fourier transform *g*(*f*_*x*_, *f*_y_) ≠ 0 for –*B*_*x*_ < *f*_*x*_ < *B*_*x*_ and –*B*_*y*_ < *f*_*y*_ < *B*_*y*_ and *g*(*f*_*x*_, *f*_y_) = 0 elsewhere (*f*_*x*_ and *f*_*y*_ for spatial frequency in the Fourier plane) and when the object is sampled at a period of *Λ*_*x*_ and *Λ*_*y*_ in the *x* and *y* axis of the object plane if *Λ*_*x*_ ≤ 1/2*B*_*x*_ and *Λ*_*y*_ ≤ 1/2*B*_*y*_. Deconvolution to obtain *f*(*x, y*) is equivalent to interpolating sampled points with a sinc function that is the Fourier-transform of the transfer function *H*(*f*_*x*_*, f*_*y*_) of a low-pass spatial filter shown in Eq. (A4). In practice, *f*(*x, y*) is not band-limited. The highest spatial frequency in the Fourier plane that is reconstructed is then determined by sampling period *Λ*_*x*_ and *Λ*_*y*_. The spatial frequency component higher than 1/2*Λ*_*x*_ and 1/2*Λ*_*y*_ along each axis causes aliasing which is, in fact, impossible to avoid in practical imaging systems. However, observable maximum spatial frequency of conventional optics is limited roughly by the cut-off frequency of the modulation transfer function (MTF) of an optical imaging system^28^. The bands that fall outside B_x_ and B_y_ of neighboring bands overlap with the main band, the effect of which can be insignificant under reasonable signal-to-noise environment.

### Sampling based on unit pulses

Sampling based on a delta comb is only conceptual. In this section, we use a more realistic sampling comb for imaging environment. Initially, suppose that the sampling comb is based on a unit pulse. Then, a delta comb is slightly broadened such that


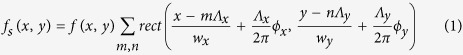


Here, *w*_*x*_ and *w*_*y*_ are the widths of the sampling function along the x and y axis that span a lateral plane. *Λ*_*x*_ and *Λ*_*y*_ represent the period in the respective axis. The widths along each axis are smaller than the sampling period, so that adjacent sampling functions do not overlap each other. *ϕ*_*x*_ and *ϕ*_*y*_ denote sampling phase between 0 and 2π, which describes displacement within a period. For simplicity, we assume that *ϕ*_*x*_ = *ϕ*_*y*_ = 0 and the effect of sampling phase will be investigated in the next chapter. Use of a unit step comb may affect the result in the way that the magnitude of a delta comb is modulated by *sinc* function, and thereby high frequency information which is generated by adjacent delta affects differently compared to an ideal delta comb. From Eq. (1), Fourier transform of a sampled object is given by


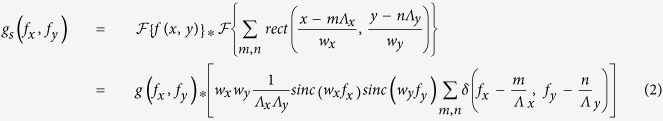


Eq. (2) can be simplified if 2D harmonic coefficients *C*(*m, n*) are introduced as


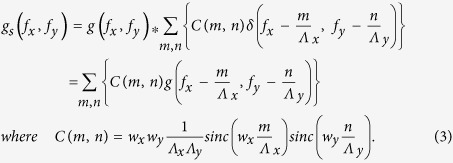


After spatial filtering, one can obtain


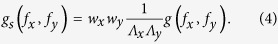


**Figure 1:**
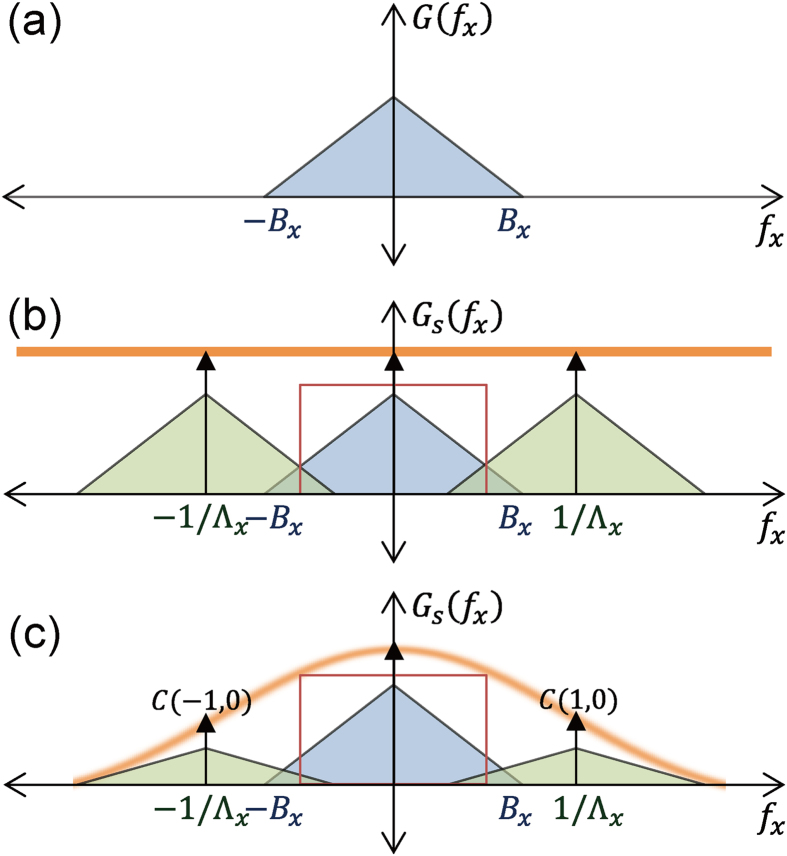
One-dimensional illustration of spatial frequency spectrum. (**a**) original object *f*(*x*) (blue triangle) which is band-limited at –*B*_*x*_ < *f*_*x*_ < *B*_*x*_, (**b**) sampled object *f*_*s*_(*x*) by an ideal delta comb, and (**c**) object *f*_*s*_(*x*) sampled by unit pulse. Green triangle and red filter represent adjacent harmonic frequency components which cause aliasing effect and low-pass filter *H*(*f*_*x*_) used for image reconstruction. With unit pulse sampling, adjacent harmonic components are reduced in amplitude by the scale factor *sinc(w*_*x*_*f*_*x*_*)sinc(w*_*y*_*f*_*y*_). Note that the orange line in (**b,c**) shows the scaling factor.

Details of the calculation are provided in Appendix B. Eq. (3) implies that the use of a unit pulse comb for sampling may in fact be desired if baseband (*m* = *0, n* = *0*) is much larger than the adjacent subbands (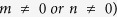, which can reduce the aliasing effect in image reconstruction, i.e., Fig. 1a–c show frequency spectrum of an object and image after sampling with a delta comb or unit pulses, respectively, in the presence of aliasing effect. If the width of unit pulses are appropriately adjusted, it is clear that the harmonic coefficients *C(m, n)* which follow *sinc* function can suppress higher-order harmonic components. On the other hand, when sampling with a delta comb as shown in Fig. 1b, adjacent subbands can overlap the baseband with the same magnitude, which may affect high frequency components of the baseband. Thus, it is expected that aliasing is more serious if an object is sampled with a delta comb than with unit pulses. This result suggests that an ideal delta comb may not be desirable for sampling: rather, a sampling function with a finite width can be desired in localization-based microscopy. If we extend this concept to sampling with an arbitrary comb other than a delta and a unit pulse, the effect will appear as a scale factor that is the Fourier-transform of a specific sampling field. Details of the sampling based on the arbitrary sampling comb are presented in Appendix C. Exact reconstruction of an object is possible as long as the shape of a sampling comb can be known in the near-field under the assumption (AS2).

We have reviewed image reconstruction and related issues in the course of sampling with a delta comb and unit pulses. The image resolution of localization-based microscopy may be affected by various parameters of sampling. This will be explored in Results and Discussion. For simplicity, we initially use unit pulses and then, for the analysis that follows, surface-enhanced near-fields.

## Results and Discussion

### Image reconstruction using unit pulses

**Figure 2:**
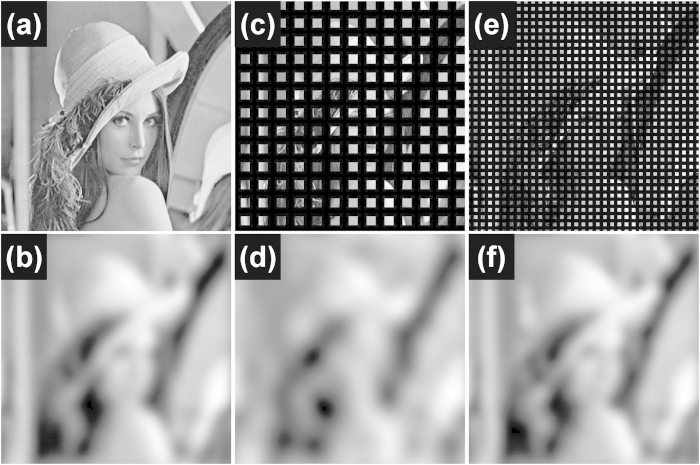
(**a**) The original object. (**b**) The image acquired by a diffraction-limited system (NA = 1.49 and λ = 500 nm). (**c**) The image sampled by a unit pulse at *Λ* = 200 nm and *w* = 100 nm with N-SLM, and (**d**) the image after low-pass filter. (**e**) The image sampled by a unit pulse at *Λ* = 80 nm and *w* = 40 nm using N-SLM and (**f**) its low-pass filtered image.

The original object and the image taken by the diffraction-limited system (NA = 1.49 and λ = 500 nm) are presented in Fig. 2a,b. For demonstration of image sampling under the diffraction-limit, first assume an image sampled by a unit pulse with 200-nm period and 100-nm width. Figure 2c exhibits an image multiplied by the unit pulse following Eq. (1), in which the pixel intensity is modulated by the pulse. To introduce degradation due to an objective lens, an image modulated by the pulse was Fourier-transformed as shown in Eq, (B3) and then multiplied by the MTF in the Fourier domain. The final image after filtering through a low-pass filter, which removes sampling artifacts under the Nyquist-Shannon sampling theorem, is shown in Fig. 2d. It is clear that the degradation in the image is such that it is difficult to visually recognize the object (Fig. 2a). For localization-based microscopy, conventional image resolution based on Rayleigh criterion is difficult to define. Therefore, for quantitative evaluation of the image reconstruction and as a measure of image resolution, we have defined a peak signal-to-noise ratio (PSNR) as^29^


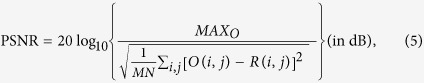


where *O(i, j)* and *R(i, j)* denote the object and the reconstructed image. *MAX*_*O*_ represents the maximum digitized field intensity value of the object image, i.e., 255 for 8-bit digitation. *M* and *N* represent the number of pixels included in the computation along the x and y axis. *i* and *j* are the pixel index on each axis. PSNR obtained by the image sampling shown in Fig. 2d was calculated to be 16.1 dB, which is much worse than PSNR = 18.1 dB for the conventional diffraction-limited image of Fig. 2b, because the maximum spatial frequency governed by the period of unit pulses is not sufficient to sample the object without aliasing and thus the sampling does not improve the image quality in this particular example.

Fig. 2e,f shows the sampled and reconstructed image with unit pulses of 80-nm period and 40-nm width. A shorter period enables the maximum spatial frequency of a low-pass filter, (1/2*Λ*_*x*_ = 6.25/μm, 1/2*Λ*_*y*_ = 6.25/μm) to become higher than the cutoff frequency of MTF (=5.96/μm). PSNR of Fig. 2e was obtained as 18.0 dB, a 1.9-dB enhancement compared to what we observed in Fig. 2d, which is almost identical to that of a conventional diffraction limited image (Fig. 2b).

**Figure 3:**
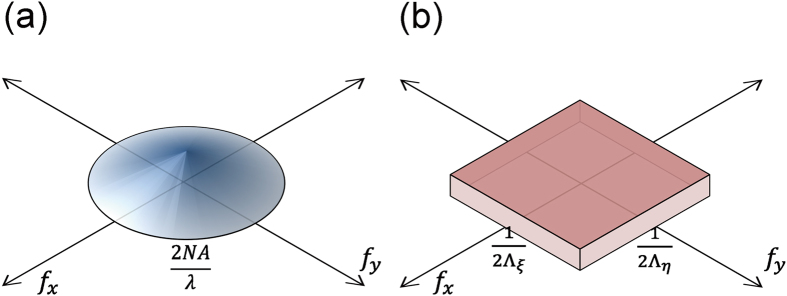
(**a**) Three-dimensional representation of MTF under incoherent illumination in diffraction-limited imaging system, in which the cutoff frequency depends on incident light wavelength and numerical aperture. (**b**) Three-dimensional representation of a low-pass filter with cutoff frequency at 1/2*Λ*_*x*_and 1/2*Λ*_*y*_.

The results can be interpreted as follows: an incoherent fluorescence imaging system with a cone-shaped MTF (shown in Fig. 3a) based on (AS1), the cut-off frequency is given by 2NA/λ (NA: numerical aperture of an objective lens) if the system is diffraction-limited. On the other hand, the frequency spectrum of a localization-based imaging system is affected by the frequency characteristics of a low-pass filter in Eq. (A4) shown in Fig. 3b. While low-pass filtering is an inevitable process to suppress sampling artifacts during image reconstruction, it is suggested that the frequency spectrum of a low-pass filter, even if it is sufficiently wider than the diffraction-limited cut-off frequency, may not improve the overall resolution of an imaging system. In other words, the final image is not limited by the maximum spatial frequency of a low-pass filter; rather it is the MTF cutoff frequency under the diffraction limit. Therefore, an extremely short sampling period may not improve the image quality and the final image can only be as good as diffraction-limited. Note that even when optical sampling is performed with a fairly long period, molecular detection may be possible at super-resolved precision if the near-field distribution of an individual localized field is precisely known (AS2). In this case, however, effective resolution obviously depends on the near-field and full-field super-resolution microscopy is not feasible.

**Figure 4:**
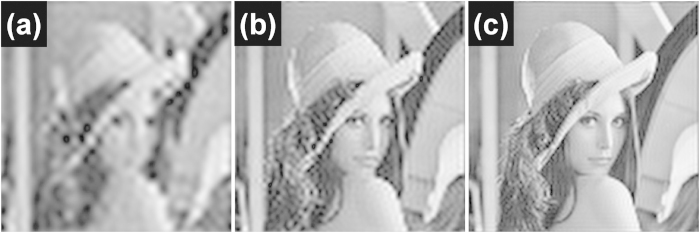
The image acquired by S-SLM with sampling parameters: (**a**) *Λ* = 80 nm and *w* = 40 nm, (**b**) *Λ* = 40 nm and *w* = 20 nm, and (**c**) *Λ* = 25 nm and *w* = 20 nm.

For this reason, in contrast to non-switchable localization microscopy (N-SLM) that is described above as based on near-field light pulses switched simultaneously, we propose spatially switchable localization microscopy (S-SLM) where an individual light pulse can be modulated by spatial switching in the near-field regardless of the period. An example of this concept is an approach attempted in plasmonic light switching^30^,^31^ and in nanoscale optical trapping^32^,^33^. In general, spatial switching of light waves on a nanometer scale remains challenging: yet, many approaches have emerged to this goal with notable success, especially in regard to temporal resolution^34^,^35^,^36^. In addition, selective switching of near-fields was attempted thorough coupling of Gaussian beams and nanoantennas^37^. S-SLM can be regarded as a super-resolution technique in the spatial domain analogous of various temporal switching techniques based on photo-switchable fluorescent probes^2^,^38^,^39^,^40^. To illustrate the concept of S-SLM in achieving super-resolution, assume a unit pulse with 80-nm period and 40-nm width (same as those used in Fig. 2e,f). Instead of switching all pulses on simultaneously, each pulse in S-SLM can be activated in a regular time interval. When a pulse is switched on, despite the spread by the diffraction under the MTF of an objective lens, the image at the imaging sensor can be reconstructed based on the shape of an activated area known *a priori*. Blurred artifact outside the excited field during temporal sampling can be removed to improve the resolution, i.e., at every interval, blurry pixels that do not overlap with the sampling field were removed as zero. By repeating the process in which all pulses at the field of view are sequentially excited to form individual images, an overall sampled image can be obtained as in Fig. 4a. Despite better resolution than the diffraction limit with PSNR = 19.6 dB, the image still suffers from ringing artifacts and the pixelated nature. The ringing is caused by high frequency aliasing and may be overcome by an anti-aliasing filter. If no Fourier plane is assumed between objective lens and object which is typical in high-magnification microscopy, ringing artifact is unavoidable. The ringing can be reduced by decreasing the sampling period, as shown in Fig. 4b. If the sampling period can be reduced to 40-nm with unit pulses of 20-nm width, PSNR improves significantly to 22.8 dB, as shown in Fig. 4b, and the reconstruction can effectively restore the original object of Fig. 3a with a sampling period of 25 nm (Fig. 4c) in which case PSNR = 25.6 dB.

The effect of sampling period and pulse width of a unit pulse comb on the image reconstruction was investigated more comprehensively, assuming symmetrical pulses in the lateral plane, i.e., *Λ*_*x*_ = *Λ*_*y*_  ≡ *Λ* and *w*_*x*_ = *w*_*y*_   ≡  *w*, for a range of 10 nm ≤ *Λ* ≤ 300 nm and 5 nm ≤ *w *≤ 295 nm in a step of 5 nm. To consider the random nature of sampling displacement of unit pulses within a period, the sampling phase ϕ was randomized in 0 ≤ ϕ < 2π with a π/2 step along x and y axis, i.e., a period is divided into 4 fragments along each axis and phases were obtained from 16 possible locations of unit pulses, whereby average and standard deviation were calculated. Note that the width cannot be larger than the period (*w* < *Λ*) for both N-SLM and S-SLM.

**Figure 5:**
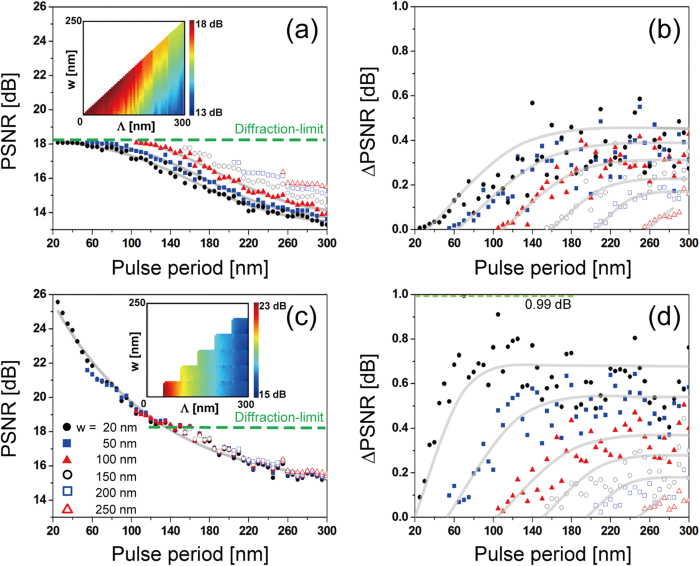
Characteristics of sampling with unit pulses as the pulse period (*Λ*) is varied. (**a**) average PSNR for N-SLM with underlying curves fitted in sigmoids. Dashed line represents PSNR corresponding to the diffraction limit and indicates that N-SLM cannot outperform a diffraction-limited system. Inset shows a complete PSNR map with period (*Λ*) and width (*w*). (**b**) The standard deviation of PSNR (ΔPSNR), which is associated with samling phase, for N-SLM. Underlying curves suggest overall trends. (**c**) Average PSNR for S-SLM and (**d**) its standard deviation. Also, underlying curves are used to suggest overall trends. For both N-SLM and S-SLM, longer periods tend to increase ΔPSNR. In S-SLM, PSNR can exceed that of diffraction-limit and is little affected by the pulse width. Inset shows a complete PSNR map interpolated with period (*Λ*) and width (*w*).

Figure 5a presents PSNR for N-SLM that was averaged over the range of sampling phase and shows that PSNR is strongly dependent on the sampling period (*Λ*). PSNR increases as the period decreases and eventually saturates with PSNR_max_ = 18.1 dB, which implies that image reconstruction may not outresolve the diffraction-limit. Put otherwise, as the sampling period increases, PSNR degrades monotonously from what corresponds to the diffraction limit. In the saturation region where PSNR coincides with diffraction-limited PSNR, maximum PSNR of N-SLM under the given wavelength is set by the MTF cutoff frequency (=5.96/μm), as mentioned earlier. To a lesser degree, PSNR also depends on the width of a unit pulse (*w*), i.e., a longer pulse increases PSNR due to reduced aliasing. Inset of Fig. 5a shows a PSNR map with respect to the period and the width of unit pulses and clearly confirms higher PSNR for a shorter period and a longer width approaching the period. Figure 5b shows standard deviation in PSNR (ΔPSNR) as the sampling phase (ϕ) is varied. Considering that the sampling phase is practically difficult to control even if the shape of unit pulses is fixed, the deviation represents unpredictability of PSNR. Although the general characteristics of the deviation look complicated, it decreases with a shorter period and a longer width, i.e., higher PSNR tends to be accompanied by smaller deviation with the maximum deviation at 0.63 dB.

For S-SLM, much different nature is observed, as shown in Fig. 5c. Most notably, PSNR is a strong function of pulse period in such a way that a shorter period increases PSNR and can be enhanced significantly beyond the diffraction limit. In contrast, pulse width affects PSNR little. The dashed line represents the diffraction limit and is obtained at around *Λ* = 155 nm. The highest PSNR was obtained as 25.6 dB at *Λ* = 25 nm. In other words, if an individual pulse can be switched, PSNR is improved by 7.5 dB or 2.37 times, compared to diffraction-limited imaging, i.e., achievable PSNR is much higher by switching and decreases quickly to the PSNR without switching for long sampling periods. Considering that PSNR is an indirect measure of resolution, the result implies that effective resolution is enhanced from 170 nm (=λ/2NA) to 72 nm (=170 nm/2.37), i.e., S-SLM can overcome Abbe diffraction limit. Although the increase of PSNR with a shorter period may look similar to what was observed in Fig. 5a, the increase is much steeper. In S-SLM, despite minor image degradation by limited MTF passband, the low-pass filter in Eq. (A4) plays a major role so that image resolution is mostly governed by the sampling period. In conventional microscopy, however, the cutoff frequency of MTF and the Rayleigh criterion have an inverse relationship stipulated by Fourier transforms^28^ and the imaging resolution is fixed by the numerical aperture. The point spread function in S-SLM is inversely proportional to the cutoff frequency of the low-pass filter, which lays the basis for the increase of resolution when the sampling period is reduced. PSNR is enhanced much more efficiently as the period becomes shorter. In addition, ΔPSNR tends to grow with a longer period in S-SLM, as presented in Fig. 5d. This may be an artifact due to the fixed number of sampling phase ϕ regardless of the period. ΔPSNR is larger in S-SLM than in N-SLM with the maximum deviation reaching 0.99 dB, which is still significantly smaller than the achievable PSNR_max_. Larger ΔPSNR in S-SLM is associated with higher sensitivity to the sampling phase. In comparison, the effect of sampling phase is drowned by the diffraction-limited MTF in N-SLM and thus causes smaller deviation in PSNR.

**Figure 6:**
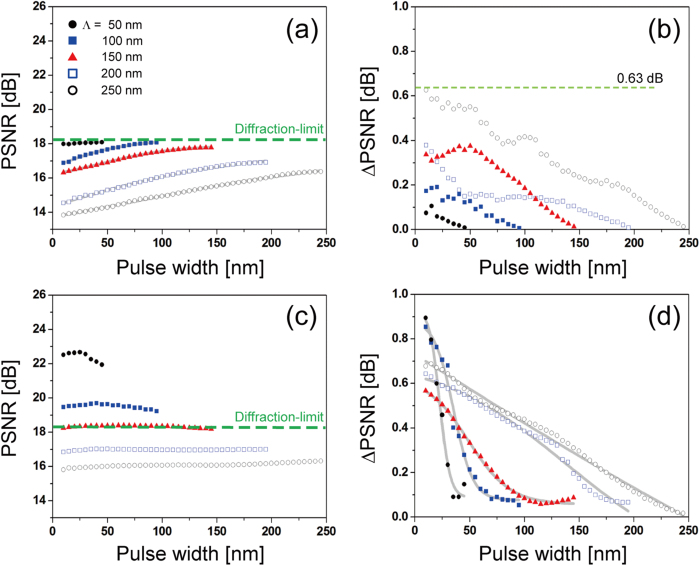
Characteristics of sampling with unit pulses as the pulse width (*w*) is varied. (**a**) average PSNR and (**b**) ΔPSNR for N-SLM. (**c**) Average PSNR and (**d**) ΔPSNR for S-SLM. Un**d**erlying curves were fitted in sigmoids. For both N-SLM and S-SLM, longer widths reduce ΔPSNR.

Figure 6 provides the dependence of PSNR on the pulse width. Without switching in N-SLM, PSNR increases with a longer pulse width. This is expected from Sec 2.2 due to the reduced aliasing effect which is caused by the presence of a scale factor, *sinc(w*_*x*_*f*_*x*_*)sinc(w*_*y*_*f*_*y*_), as shown in Fig. 6a. The deviation clearly decreases with a longer width in Fig. 6b. The ratio of the pulse area to the total sample area becomes higher with a longer pulse width, thus the variation in the sampling location, which is represented by the fluctuation in sampling phase, shows less pronounced effect. Therefore, uncertainty is reduced with a longer pulse width. On the other hand, PSNR is little affected by the pulse width for S-SLM (Fig. 6c), i.e., from linear fits, the PSNR change per unit aperture length is given by PSNR/L = 0.0007, 0.0001 and 0.0014 dB/nm respectively for Λ = 150, 200, 250 nm. For comparison, PSNR/L = 0.0114, 0.0132 and 0.0113 dB/nm in N-SLM for the same periods (Fig. 6a). This emphasizes much less influence of the pulse width on PSNR (thus resolution) in S-SLM, particularly in the case of a long sampling period. In S-SLM, aliasing does not occur because of the lack of crosstalk between neighboring samples even at high spatial frequency. ΔPSNR shown in Fig. 6d also decreases with a longer pulse width and the decrease is much more drastic for pulses with a shorter width.

Summarizing this section, localization microscopy can achieve super-resolution based on switching of individual unit pulses for S-SLM. The achievable PSNR in the range of pulse widths and periods was obtained as 25.6 dB, 7.5 dB higher than the diffraction limit.

### Image reconstruction using surface-enhanced localized near-fields

**Figure 7:**
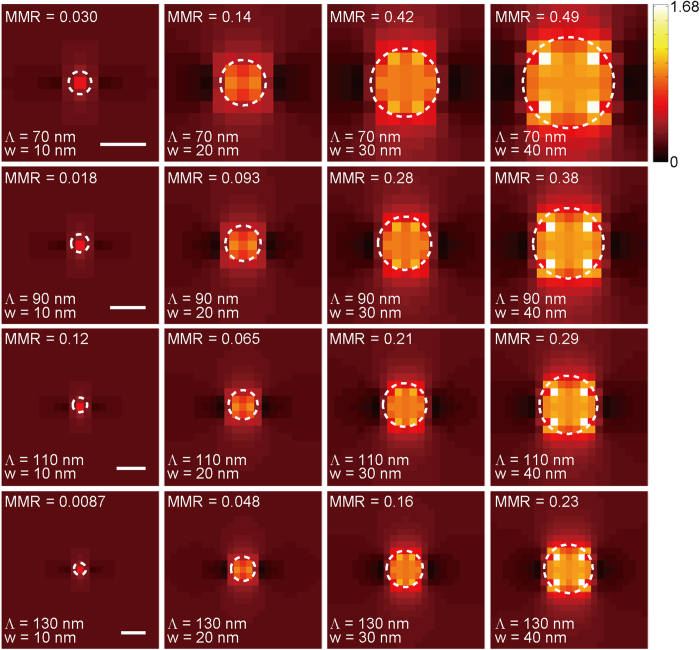
Near-field distributions that are produced by nanoholes (*w* = 10, 20, 30 and 40 nm & *Λ* = 70, 90, 110, and 130 nm). Dashed circles represent the nanohole apertures. Scale bar: 20 nm. Scale bar is identical in rows. MMR for each nanohole aperture pattern is also presented.

We now consider actual near-fields produced by surface nanohole arrays as the comb that excites fluorescence for far-field images. The main difference from the case of using unit pulses is the shape in the near-field as well as the presence of side modes and background that may create additional noise and cause PSNR to further degrade. For calculation, the aperture period (*Λ*) between nanoholes was varied from *Λ* = 40 to 250 nm with a 10-nm interval. At each period, the nanohole diameter or aperture width (*w*) was varied from 10 nm to a value that is 30 nm less than the period, i.e., 10 nm ≤ *w* ≤ *Λ* − 30 nm, with an interval of 10 nm, i.e., the calculation was performed in a range narrower than the case of unit pulses. Figure 7 presents the near-field distribution for select 16 combinations of *Λ* and *w* among total 300 cases that were calculated. To assess the quality of localized near-fields produced by each aperture on a quantitative basis, we have defined a main-mode ratio (MMR) as the ratio of optical power contained in the main peak whose relative field intensity exceeds 0.5 to the total optical power, i.e.,


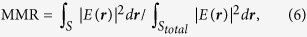


where *S* is a subset area where |*E(**r**)*|^2^ > 0.5 and the integral in the denominator is performed across the total area (*S*_*total*_). MMR represents how much power is concentrated in the localized spot and can be used as a simple metric of the degree of field localization. For unit pulses, field intensity at the spot is equal to unity while it is zero outside the pulse, therefore MMR = 1. MMR for localized fields becomes less than unity because of non-zero field distribution in the background. Calculated MMR appears in Fig. 7 for the presented nanohole patterns.

**Figure 8:**
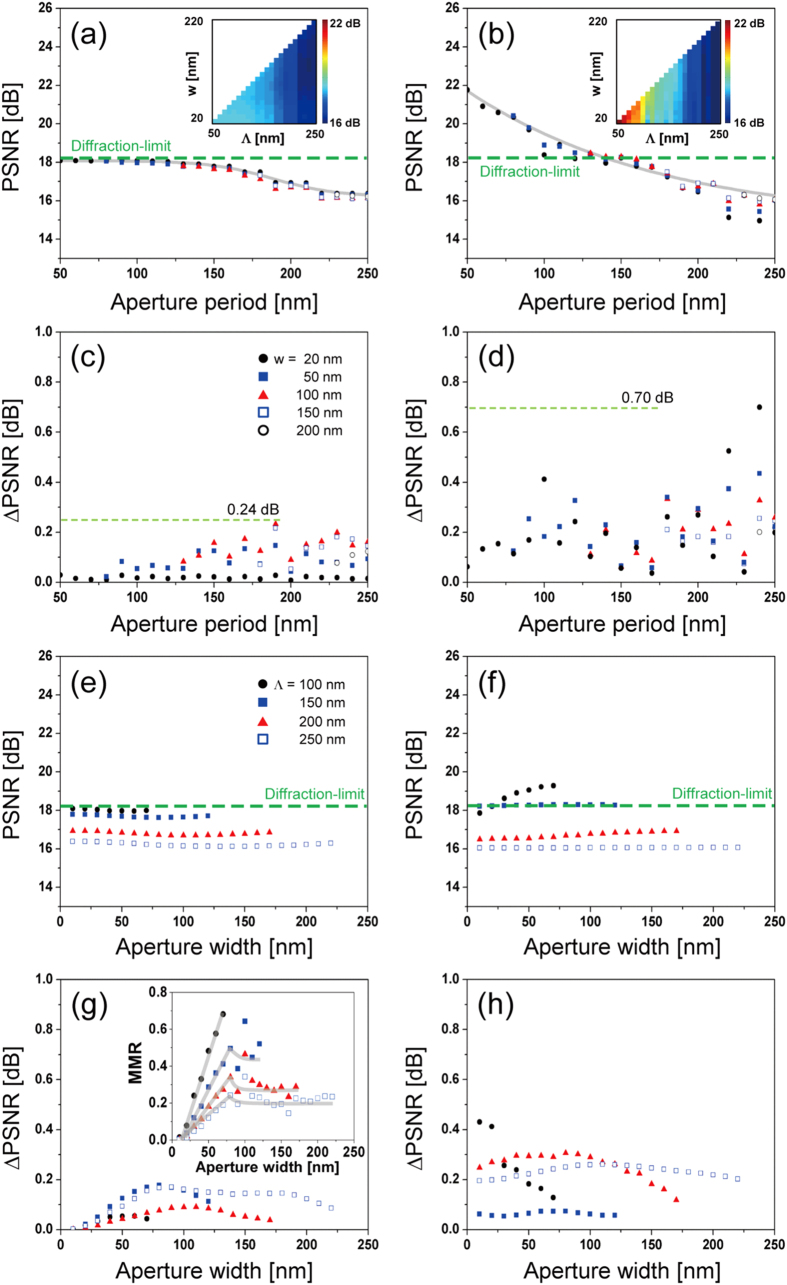
Characteristics of sampling with surface-enhanced near-fields localized by arrays of nanoholes as the aperture period (*Λ*) and width (*w*) are varied: average PSNR for (**a**) N-SLM and (**b**) S-SLM with respect to the period (*Λ*). Underlying curves were fitted in sigmoids and the dashed line represents PSNR corresponding to the diffraction limit. Insets show a complete PSNR map with period (*Λ*) and width (*w*). ΔPSNR for (**c**) N-SLM and (**d**) S-SLM. Average PSNR for (**e**) N-SLM and (**f**) S-SLM with respect to the aperture width (*w*). ΔPSNR: (**g**) N-SLM and (**h**) S-SLM. Inset in (**g**) shows the MMR which represents the degree of field localization produced by each aperture.

If we compare PSNR, the overall behavior is similar to what was observed by sampling with unit pulses for both N-SLM and S-SLM, as shown in Fig. 8a,b, i.e., a shorter aperture period between localized fields increases PSNR. Similar to the case of using unit pulses, blurred image artifact outside the main mode was removed for improvement of image quality. In S-SLM, the achievable PSNR decreases when surface-enhanced localized field is employed: for example, at *Λ* = 50 nm and *w* = 20 nm, PSNR = 21.5 dB in contrast to 22.6 dB in the case of using unit pulses with identical sampling parameters. 1.1 dB (=22.6 − 21.5 dB) penalty is presumably associated with reduced MMR due to the presence of side modes and background that interfere with the main mode of field localization. ΔPSNR is smaller than using unit pulses for both N-SLM and S-SLM, as shown in Fig. 8c,d. The maximum PSNR and ΔPSNR that were calculated under various scenarios are summarized in Table 1. Reduced ΔPSNR is attributed to sampling pulses being larger, less abrupt, and more continuous in nature when near-fields are localized than unit pulses. Therefore, the variation that the combinations of different sampling positions and sampling phase give rise to becomes smaller, i.e., localized fields with a lower MMR may lead to reduced ΔPSNR. In other words, if pulse width is significantly smaller than the difference between sampling positions, the variation can be proportionately larger. Effect of polarization was also checked with circularly polarized light incidence: preliminary data suggest that PSNR changes little while ΔPSNR is slightly reduced compared to that of linear polarization (data not shown).

PSNR is little affected by the aperture width (Fig. 8e,f), which agrees well with the behavior shown with unit pulses in Fig. 6a,c. One major difference with using localized fields is the deviation with respect to the aperture width shown in Fig. 8g,h. The results indicate the existence of a width at which the deviation reaches a maximum, rather than a monotonous decrease of ΔPSNR with a longer width which we observed with unit pulses. The inset of Fig. 8g which shows the MMR with respect to aperture width strongly suggests that the trend of ΔPSNR should be closely correlated to MMR. In this sense, ΔPSNR can be regarded as an indicator of near-field localization mentioned earlier.

The results of using localized near-fields for sampling suggest that the imaging characteristics should be largely consistent with the case of unit pulses. Super-resolved data can be extracted using localized light fields by switching in S-SLM, the precision of which is dominated by the degree of localization. Although there is penalty in the achievable PSNR (approximately 1 dB under identical sampling parameters) compared to unit pulse sampling, super-resolution may be obtained for full-field microscopy. Highest PSNR was calculated to be 22.0 dB in this case, i.e., 3.9 dB enhancement implies effective resolution on the order of 108 nm in the range of parameters that we considered when sampling with fields localized by nanohole apertures. The penalty may be reduced or even avoided if apertures that are more efficient in localization than nanoholes, such as nanogaps, are employed. Spatial localization as a result of various apertures was described elsewhere^17^.

## Discussion

We have found the highest effective resolution on the order of 70 nm using unit pulses and 100 nm with localized fields. Obviously, a shorter pulse period can lead to a higher resolution: if we estimate the feature size in commercially available fabrication to lie between 10 and 20 nm, the highest resolution that is practically meaningful may be under 50 nm. With a nanostructure on a subnanometer scale, quantum effect kicks in and may nullify assumptions made in this study. With 1-nm presumed as the ultimate period, PSNR ≈ 39 dB, which corresponds to 15 nm as the highest resolution theoretically possible.

What was found in this study shows both strengths and weaknesses of full-field nanoscopy based on near-field super-localization. Above all, it was found that full-field super-resolution can be achieved by spatial switching of localized fields. Main determinant of the resolution is the period between sampling fields, which in fact reflects the Nyquist-Shannon sampling theorem. The super-localization technique that we investigated here has the scanning nature in common with other spatial sampling approaches including STED microscopy, which relies on fluorescence emission and depletion to reduce point spread and scans the reduced PSF. This is also true with the methods such as PALM or STORM which sample in the time domain rather than spatially. Depending on the specific approaches available for localizing near-field and switching it, S-SLM can be an alternative to the established super-resolution microscopy techniques.

## Summary

We have explored how to achieve super-resolution in localization-based microscopy. The results suggest that images at super-resolution beyond the diffraction limit can be obtained by spatial switching of localized sampling fields for S-SLM. For comparison, N-SLM is much easier to implement, which cannot outresolve the diffraction limit. For the range of periods and widths of sampling pulses that we considered here, it was found that the period is the main factor that determines resolution and should be shorter than 150 nm to attain super-resolution. Use of near-fields localized by periodic nanoapertures instead of ideal unit pulses for sampling suffers from penalty in the achievable resolution by less than 1-dB in terms of PSNR. The penalty is associated with side modes and background present in surface electromagnetic wave distribution. Highest resolution that may be achievable under full-field microscopy is estimated as 70 nm with unit pulses which may be further enhanced by optimizing sampling parameters.

## Methods

### Image reconstruction

For quantitative evaluation of image reconstruction in localization based microscopy, a standard gray scale image of Lena in 512 × 512 pixels was used as an object, where the size of a pixel is assumed to be 5 × 5 nm^2^, i.e., the test image is as large as 2.56 × 2.56 μm^2^. All images under comparison are normalized in intensity to ensure that the image quality should not be affected by image brightness.

The image reconstruction using unit pulse combs was calculated by Matlab™ in custom-built codes. For image reconstruction based on surface-enhanced field localization, near-field calculation was performed in 3D by finite difference time domain method (mesh size: 5 × 5 nm^2^) assuming 488 nm as the excitation wavelength. The model for surface nanoapertures that are used to localize light waves consists of a gold film (thickness = 52 nm) patterned with nanohole arrays and a 2-nm thick chromium adhesion layer on a BK7 glass substrate. The nanoholes used in the model were 50 nm deep. A linearly polarized wave was assumed to be normally incident with periodic boundary conditions. The electric field intensity distribution was calculated in the lateral plane at the middle of a nanohole along the depth axis.

## Additional Information

**How to cite this article**: Son, T. *et al.* Localization-based full-field microscopy: how to attain super-resolved images. *Sci. Rep.* **5**, 12365; doi: 10.1038/srep12365 (2015).

## Figures and Tables

**Table 1:** Maximum PSNR and ΔPSNR obtained by N-SLM and S-SLM based on sampling with unit pulses and localized fields.

	**PSNR_max_ (dB)**		**ΔPSNR_max_ (dB)**	
	**N-SLM**	**S-SLM**	**N-SLM**	**S-SLM**
Unit pulse sampling	18.1 (*Λ* = 20 nm and *w* = 10 nm)	25.6 (*Λ* = 25 nm and *w* = 20 nm)	0.63 (*Λ* = 250 nm and *w* = 10 nm)	0.99 (*Λ* = 70 nm and *w* = 20 nm)
Sampling with localized fields	18.1 (*Λ* = 90 nm and *w* = 10 nm)	22.0 (*Λ* = 40 nm and *w* = 10 nm)	0.24 (*Λ* = 190 nm and *w* = 120 nm)	0.69 (*Λ* = 240 nm and *w* = 20 nm)

The presented PSNR and ΔPSNR only reflect the results in the range of sampling parameters that were considered. Parentheses are the sampling parameters with which specific PSNR and ΔPSNR were obtained.
